# Challenges when measuring person-centred dementia care in nursing homes: a discussion paper

**DOI:** 10.1016/j.ijnsa.2026.100638

**Published:** 2026-07-20

**Authors:** Anna Louisa Hoffmann-Hoffrichter, Rebecca Palm, Bernhard Holle, Martina Roes

**Affiliations:** a German Center for Neurodegenerative Diseases (DZNE), site Witten, Stockumer Str. 12, 58453, Witten, Germany; b Witten/ Herdecke University (UW/H), Faculty of Health, Department of Nursing Science, Alfred-Herrhausen-Straße 50, 58448, Witten, Germany; c Carl von Ossietzky Universität Oldenburg, School VI – School of Medicine and Health Sciences, Ammerländer Heerstraße 114-118, 26129, Oldenburg, Germany

**Keywords:** Person-centred care, Person-centredness, Dementia, Measurement, Dementia care research

## Abstract

The gold standard of person-centred dementia care in nursing homes is required by international guidelines. Nevertheless, the implementation of this approach remains challenging. Measurements designed to observe outcomes associated with the implemented intervention, assess the attitudes of relevant audiences, investigate the effectiveness of person-centred care interventions, and evaluate the extension of person-centred care implementation in long-term care practice are necessary to overcome these challenges. Nonetheless, capturing the core of person-centredness remains challenging in implementation and intervention studies. For this discussion paper, we conducted a literature search to address three different perspectives on the measurement of person-centred care in the context of its implementation into practice: (1) conceptual, (2) interventional, and (3) organizational perspectives. This paper aims to provide insights into measurements that have been used in dementia care research and to discuss the challenges that correspond with measuring person-centred dementia care in nursing homes. Based on the discussion, we provide recommendations for future measurements of person-centred care in the conclusion section. Our recommendations address key aspects that we deem necessary for an adequate person-centred care measurement in the future, including (1) a consensus on a definition of core elements of person-centred dementia care and the provision of adaptable person-centred dementia care-related concepts and (2) multidimensional, (3) multiperspective, and (4) multimethodological approaches that aim to capture the core and adaptable elements of person-centred care. All key aspects are consistent with and are influenced by the engagement of people living with dementia, their relatives, and relevant stakeholders as well as a continuous, transparent and joint dialogue on every key aspect.

**What is already known**

• Various measurements exist in dementia care research to assess person-centred care.
• There is no existing measurement that measures person-centred care holistically.

Alt-text: Unlabelled box dummy alt text

**What this paper adds**

• A transparent overview of challenges in measuring person-centred dementia care.
• Suggestions framed as recommendations for future measurements of person-centred care.
• A suggestion of a new approach to measure person-centred care.

Alt-text: Unlabelled box dummy alt text

## 1 Introduction

The predicted prevalence of dementia in Europe for 2025 is 10.3 million people (Alzheimer Europe, 2019), resulting in a social requirement to enable people with dementia to continue to live an autonomous and a satisfactory life. Particularly when it is no longer possible to care for people living with dementia at home, this requirement presupposes care structures in nursing homes that are characterized by professionalism, respect, understanding and empathy (Bundesministerium für Familie, Senioren, Frauen und Jugend (BMFSFJ) & Bundesministerium für Gesundheit (BMG), 2020). Moreover, the World Alzheimer’s Report (Alzheimer's Disease International, 2024) highlights attitudes towards dementia, where people living with dementia describe themselves as experiencing discrimination (Alzheimer's Disease International, 2024). With respect to healthcare staff, people living with dementia experience treatments in paternalistic ways, which are characterized by loss of autonomy or exclusion (Eriksen et al., 2016).

Kitwood (1997) introduced the concept of person-centred care to the care of people living with dementia for the first time to move away from this so-called malignant social psychology that accompanies people living with dementia where their personhood is undermined by others and by being pathologized. In this article, we address this approach as person-centred dementia care. Person-centred care is a popular approach (World Health Organization, 2015) and is fundamental in long-term residential care for people living with dementia (Fazio et al., 2018). This approach focuses on the person and aims to maintain selfhood and identity (Fazio et al., 2018). Brooker and Latham (2015) defined key components of person-centred dementia care, the so-called VIPS: V for valuing the person living with dementia and their carers, I for the individual treatment of the person living with dementia who has unique needs, P for a consideration of the perspective of the person living with dementia and S for the social environment that is relevant to experience care that promotes relationship building.

In person-centred dementia care research, person-centred care can be implemented as a whole concept (Edvardsson et al., 2008; Lee et al., 2023), as single person-centred care interventions (Blake et al., 2020; Saragih et al., 2025), or as organizational interventions (Quasdorf & Bartholomeyczik, 2019), such as staff training (Sefcik et al., 2022) or environmental design changes (Fahsold et al., 2024; Jutkowitz et al., 2016). Therefore, in this discussion paper, we distinguish three perspectives that are essential for person-centred care and its implementation into practice, namely: (1) conceptual, (2) interventional, and (3) organizational perspectives. We define the first perspective as the conceptual perspective, which refers to the existence of a concept for person-centred dementia care in the respective institution, and includes content about the person-centred philosophy, mindset, and culture. The second perspective, the interventional perspective, is understood as the application of specific person-centred interventions embedded in and evaluated within the nursing care process. Third, the organizational perspective refers to structures, policies, and leadership when person-centred care concepts and interventions are implemented.

Person-centred care is considered “high-quality care” (World Health Organization, 2015), but its implementation might be complex and challenging. The reasons for this challenge may include the following: (1) theoretical understandings of the person-centred care concept are heterogeneous (Serbser-Koal, Rommerskirch-Manietta, et al., 2024), and (2) the effectiveness of person-centred care depends on how person-centred care is provided quantitatively and qualitatively (Li & Porock, 2014).

Measurements with high psychometric properties are necessary to observe outcomes, assess the attitudes of relevant audiences, investigate the effectiveness of person-centred care interventions (Werner et al., 2025), and evaluate the extension of person-centred care implementation in long-term care practice (Shier et al., 2014) to overcome the challenges associated with person-centred care implementation. Nonetheless, measuring person-centredness remains challenging (Li & Porock, 2014).

In terms of which of these abovementioned perspectives are relevant when assessing person-centred dementia care within dementia care, different methodological approaches, designs, and measurements are needed. This paper aims to provide insights into measurements that have been used in dementia care research and to discuss challenges that correspond with assessing person-centred dementia care in nursing homes. In this discussion paper, we use the term “measurement” but also include assessments, tools, and instruments.

We mentioned that in person-centred dementia care research, the implementation of person-centred care can be characterized from conceptual, interventional and organizational perspectives. Therefore, in this discussion paper, we consider the mentioned (1) conceptual, (2) interventional, and (3) organizational perspectives as key perspectives when measuring person-centred care in the residential long-term care of people living with dementia and discuss them.

In accordance with the aim of this discussion paper, we searched for relevant reviews describing the different perspectives on measurement but did not aim for completeness: In October 2025, we conducted a systematic search in the Medline database via PubMed. The search terms were selected on the basis of Corlin’s approach (Elfstrand Corlin & Kazemi, 2024), using terms related to person-centred care as well as terms related to measurements. Access to the search string is possible upon request to the authors.

With respect to the inclusion criteria, we included only publications defined as review by the authors (such as systematic reviews, reviews of systematic reviews, scoping reviews, integrative reviews, comprehensive reviews, rapid reviews, state-of-the-art reviews, review articles, critical comparative reviews, narrative reviews, literature reviews, or evidence reviews) published in English or German that address person-centred measurement regarding the concept and implementation as well as person-centred interventions. To include every relevant review on person-centred care and its measurement, no time limit was set for the search.

We identified 2678 articles. The title/abstract screening of the identified articles was conducted by the author (ALH) via the systematic review platform Rayyan (2026). The title/abstract screening resulted in 88 included articles, and their full texts were subsequently screened. Neither a critical appraisal of the included articles nor a risk of bias assessment was conducted. Data extraction was conducted by the 1^st^ author (ALH) and discussed with the co-authors. We analysed the included articles regarding the measurement of person-centred care using a qualitative content analysis approach (Kuckartz & Rädiker, 2022), thus we applied a deductive approach, using the conceptual, interventional and organizational perspectives as the main categories. Afterwards, we constructed subcategories for each main category derived from the included articles.

In the following sections, we introduce each mentioned perspective by providing a short description of the respective topic. After each introduction, we describe the results of our literature search. Based on this information, we discuss the challenges associated with assessing person-centred care. We conclude this discussion paper with recommendations for the measurement of person-centred dementia care in nursing homes.

## 2 Assessing person-centred care from a conceptual perspective

With respect to the implementation of person-centred care in care practice, a person-centred care concept is necessary. The concept of person-centred care has its origin in the psychotherapy of Carl Rogers (Rogers, 1961). In the context of his client-centred approach, he addresses the process of becoming self and the required client–counsellor relationship, which is characterized by safety and freedom (Rogers, 1961). In the context of caring for people living with dementia, Kitwood (1997) first described the person-centred care paradigm. In the context of malignant social psychology, he demonstrated the discriminant process of the depersonalization of people living with dementia. The person-centred care approach focuses on the person who comes first. The aim is to maintain personhood (Kitwood, 1997), a status that is connected with emotions, feelings and power to be in relationships (Post, 2000). In this context, both Rogers (1961) and Kitwood (1997) address the fundamental work of Buber (2013) “I and Thou”, wherein personhood is standing in relation in that one is addressed as you. In addition to relationships, other key aspects of personhood are individualization and embodiment (Kitwood, 1997).

Since the study by Kitwood (1997), the person-centred care approach has been further developed into various models and frameworks. In addition to Brooker and Latham (2015) and the earlier mentioned VIPS model, concepts, models, and frameworks aiming for person-centredness that are not specific to people living with dementia have been proposed. McCormack and McCance (2021) highlighted the complexity of structures, processes, and specific outcomes in person-centred nursing in general by developing the Person-Centred Nursing Framework. As a middle range theory, this structured and practice-oriented framework consists of four core elements: prerequisites (attributes of the nurse), the care environment (in which care is provided), person-centred care processes (operationalizations of person-centred care in activities), and outcomes such as good care experience (McCormack & McCance, 2021). Nolan et al. (2006) developed the Senses Framework, which includes the relationship-centred model and focuses on a care environment where not only the person receiving care is the centre but all participants in care are addressed and acknowledged. Furthermore, concepts such as patient-centred care, which focuses on the patient (Pakkonen et al., 2021); client-centred care, which is addressed in service provider publications (Edvardsson & Innes, 2010); and individualized care, which focuses on the uniqueness of the care recipient (Edvardsson & Innes, 2010), have been proposed.

In the context of people living with dementia, concepts for person-centred dementia care in nursing homes remain deeply anchored in Kitwood’s theoretical framework (Kitwood, 1997). Nonetheless, concepts for person-centred dementia care have grown to explicitly address heterogeneous definitions of autonomy (Serbser-Koal, Dreyer, et al., 2024), social justice (Victor et al., 2024), diversity (Peters-Nehrenheim et al., 2022), and intersectionality (Altinok et al., 2025b), especially in response to multiculturalism and marginalized group needs in care settings over the past decade (Altinok et al., 2025a, 2025b; Victor et al., 2024).

The results of the literature search show that to date, various measurements have been developed to measure person-centred care, from measurements that aim to assess the concept to measurements that focus only on one aspect or subcomponent of person-centred care. Person-centred care can be measured either in an observation or in a staff or patient survey, as well as in interviews with professionals or patients, or through a patient note review (De Silva, 2014).

With respect to measurements that assess the concept of person-centred care, Dementia Care Mapping (DCM) is the only dementia-specific measurement used to assess person-centred care and was the first measurement developed to embed person-centred dementia care (University of Bradford, 2026). Originally, Dementia Care Mapping (DCM) was developed to evaluate care but was not designed for use in research. To date, this measurement has been used as a practice measurement, as an evaluation measurement for interventions, as well as an intervention and an outcome measurement (Edvardsson & Innes, 2010). The VIPS Assessment/Reflecting Tool is also used in nursing practice (Brooker & Latham, 2015). Other measurements assessing the person-centred care concept in the long-term care setting are not dementia specific but intended for research use. In our literature search, most publications addressed the Person-Centered Care Assessment Tool (P-CAT) (Edvardsson et al., 2010) as cited by Bru-Luna et al. (2024), Edvardsson and Innes (2010), Fazio et al. (2018), (Pakkonen et al., 2021), and van Diepen et al. (2020), followed by the Person-Directed Care Measure (White et al., 2008) cited by Fazio et al. (2018), and Measures of Individualized Care cited by Edvardsson and Innes (2010), Fazio et al. (2018), and van Diepen et al. (2020). Other measures assess culture change, such as the Culture Change Scale and the Culture Change Staging Tool (Grant, 2008), as cited by Shier et al. (2014).

We have also identified assessments that measure aspects or subcomponents of person-centred care with the intention of research use that we assign to the conceptual perspective: Family Involvement in Care measures the involvement in the care of persons living with dementia perceived by a family member (Reid et al., 2007), as cited by Fazio et al. (2018). The Person-Centred Climate Questionnaire (Edvardsson et al., 2009) cited by Pakkonen et al. (2021) assesses the extent of climate in health care settings and includes three different versions exploring different perspectives, such as the staff version, the patient version, and the family version. An example of the dementia-specific assessment of single aspects or subcomponents of the person-centred care approach is the Approach to Dementia Questionnaire (Lintern, 2001), as cited by van Diepen et al. (2020).

## 3 Assessing person-centred care from an interventional perspective

With respect to the implementation of person-centred care from an interventional perspective, person-centred care can be applied as a strategy within the nursing process. Strategies to provide person-centred care are person-centred care interventions (Edvardsson et al., 2008). The reviews included in the literature search address various person-centred care interventions for people living with dementia in nursing homes. These interventions include Dementia Care Mapping (DCM) (Berkovic et al., 2024; Blake et al., 2020; Saragih et al., 2025); psychosocial interventions such as music-based interventions (Berkovic et al., 2024; Lee et al., 2023; Mei et al., 2025; Saragih et al., 2025), reminiscence (Baggaley et al., 2024; Saragih et al., 2025; Wong et al., 2024), validation therapy (Blake et al., 2020; Fazio et al., 2018), arts intervention (Baggaley et al., 2024; Xu et al., 2025), or robotic care interventions (Hirt et al., 2021; Lu et al., 2021; Saragih et al., 2022); various forms of staff training (Blake et al., 2020; Pakkonen et al., 2021; Sefcik et al., 2022; Zhao et al., 2021); organizational interventions such as culture change interventions (Fazio et al., 2018; Shier et al., 2014); and individual interventions such as a review of medication use and care planning (Jutkowitz et al., 2016).

When the effectiveness of person-centred care interventions for people living with dementia is being investigated (Kim & Park, 2017; Lee et al., 2022), research teams include various outcomes and end points and therefore apply various measurements that assess these outcomes. In intervention studies, outcomes are often used as proxy descriptors of person-centredness (Edvardsson & Innes, 2010) to provide information about the effectiveness of the intervention. These outcome measurements include measures to assess the outcomes of individual residents (Baggaley et al., 2024; Couch et al., 2020; Hirt et al., 2021; Hofbauer & Rodriguez, 2025; Mei et al., 2025; Wong et al., 2024; Xu et al., 2025), staff outcomes (Berkovic et al., 2024; Cano et al., 2023; Pakkonen et al., 2021; van Diepen et al., 2020), family member outcomes (Burgers et al., 2021; Cano et al., 2023; Tu et al., 2022; Wong et al., 2024), and quality of care and service outcomes (Cano et al., 2023; Deprez et al., 2024; Pakkonen et al., 2021; Sefcik et al., 2022). The outcomes of individual residents include behaviour (Hofbauer & Rodriguez, 2025; Saragih et al., 2025; Sebalj et al., 2024; Xu et al., 2025), quality of life (Berkovic et al., 2024; Hofbauer & Rodriguez, 2025; Kenning & Visser, 2021; Lu et al., 2021; Saragih et al., 2025), cognitive function (Couch et al., 2020; Lee et al., 2022; Saragih et al., 2025; Wong et al., 2024), and behavioural and psychosocial symptoms of dementia (BPSD) (Mei et al., 2025; Saragih et al., 2025; Wong et al., 2024; Xu et al., 2025). Staff outcomes refer to competences (including communication skills, knowledge, and behaviour management skills) (Berkovic et al., 2024; Blake et al., 2020; Zhao et al., 2021); emotions (such as psychological outcomes and mood or job satisfaction) (Cano et al., 2023; Sefcik et al., 2022; Zhao et al., 2021); staff readiness (such as attitudes or self-efficacy for dementia) (Elfrink et al., 2018; Nguyen et al., 2019; Zhao et al., 2021); and psychological aspects (such as stress, burnout, depression, or burden) (Couch et al., 2020; Spector et al., 2016; Woods et al., 2018). Family member outcomes include strain (Saragih et al., 2022; Tu et al., 2022; Wong et al., 2024) or family caregiver gains (Cano et al., 2023). Quality of care and service outcomes include staff–patient/resident interactions (Blake et al., 2020; Cano et al., 2023; Pakkonen et al., 2021), acute hospital admissions (Bird et al., 2016), and restrictive practices (Bird et al., 2016; Sefcik et al., 2022).

## 4 Assessing person-centred care from an organizational perspective

With respect to the implementation of person-centred care concepts, a supportive organization is needed (Chenoweth et al., 2019). We refer to the organizational perspective as leadership, structures, and internal regulations. The leadership function is pivotal for creating a person-centred climate that includes shared governance and organizational attitudes (Hoffmann-Hoffrichter et al., 2025). Leadership also influences institutional structures, such as the facility, personnel and care structure (Hoffmann-Hoffrichter et al., 2025). Quasdorf and Bartholomeyczik (2017) reported that manager leaders must provide a vision of person-centred dementia care. This vision is also anchored in the mission statement regarding the successful implementation of this approach (Quasdorf & Bartholomeyczik, 2017). Furthermore, regulations affect the operationalization of person-centred dementia care (Ahmed et al., 2019; Santana et al., 2019), especially when such care is controlled by regulators (Pot et al., 2024). In this discussion paper, internal regulations include policies, written standards and other regulations in a nursing home.

There are various assessments that we classify as measuring an organizational perspective. A practice measurement that includes all organizational levels in the evaluation of organizational strengths and weaknesses regarding person-centredness is the VIPS framework (Brooker & Latham, 2015; Røsvik et al., 2011). The Person-Centred Practice Inventory consists of three assessments: the Person-Centered Practice Inventory–Staff (PCPI-S) (Slater et al., 2017), the Person-Centred Practice Inventory–Care (PCPI-C) (McCormack et al., 2024), and the Person-Centred Practice Inventory–Student (PCPI-ST) (O'Donnell et al., 2021). Each of the measurement surveys includes different perspectives and therefore addresses different components of the Person-Centred Practice Framework by McCormack and McCance (2017). Other measurements that were classified as measuring the conceptual perspective and mentioned above could also be assigned to the organizational perspective. These measurements include the Family Involvement in Care (Reid et al., 2007) cited by Fazio et al. (2018) and the Person-Centred Climate Questionnaire (Edvardsson et al., 2009) cited by Pakkonen et al. (2021).

In the literature search, the included reviews addressed specific outcomes from an organizational perspective that differed from study to study, regardless of the implementation of the holistic person-centred care approach or a person-centred care intervention. Organizational outcomes include the environment, nursing assistant retention, physical restraint use, costs, management, and psychotropic use, as well as an increase in person-centred care policies. Measurements such as the Mealtime Scan (Keller et al., 2018) cited by Deprez et al. (2024) or the Environment Assessment for Person-Centered Management of BPSD (Resnick et al., 2020) cited by Cano et al. (2023) were used to measure the environment. Measurements such as the Baldrige Leadership Scale cited by Spector et al. (2016), were used to assess management.

According to Chenoweth et al. (2019), two measurements are relevant to person-centred dementia care from an organizational perspective: (1) the Short Observation Framework of Inspection, version 2 (SOFI 2), which is not designed for research purposes but for Care Quality Commission (CQC) inspectors to observe staff interaction quality as well as the engagement and mood of people obtaining services (Bradford Dementia Group and the Commission for Social Care Inspection in England, 2007); and (2) the Person-Centred Environment and Care Assessment Tool (PCECAT) (Burke et al., 2016), a research tool designed to assess both service quality at the individual level and facilitating organizational elements for person-centred care.

With respect to the implementation of person-centred dementia care, one possibility when focusing on the organizational context is to concentrate on the existence of person-centred regulations for nursing homes. We identified one measurement that focuses on the implementation of policies regarding the person-centred management of behavioural and psychological symptoms of dementia (BPSD). The “Assessment of Policies of Person-centred Management of BPSD” developed by Resnick et al. (2020) aims to measure how policies on the behavioural and psychological symptoms of dementia (BPSD) influence residents of nursing homes.

Resnick’s assessment was the basis for the development of a measurement focusing on the German nursing home context named the Dementia Policy Questionnaire (DemPol-Q) (Hoffmann et al., 2022). The Dementia Policy Questionnaire is a measurement that aims to measure the existence of policies of person-centred dementia care in German nursing homes (Hoffmann et al., 2022).

## 5 Discussion of the challenges associated with assessing person-centred care

### 5.1 Challenges in assessing person-centred care from a conceptual perspective

The assessment of person-centred care from a conceptual perspective is important for confirming the successful implementation of person-centred care approaches in practice (Gschwenter et al., 2023) and for providing details about the quality of performance of person-centred care (Byrne et al., 2020). However, the literature search revealed that person-centred care is multifaceted and that the various person-centred care measurements focus on different aspects (Elfstrand Corlin & Kazemi, 2024).

A measurement to cover all aspects of the multidimensional, holistic person-centred care concept is still lacking (De Silva, 2014; Elfstrand Corlin & Kazemi, 2024). In fact, various challenges in the assessment of person-centred care from a conceptual perspective exist. The main challenges are the heterogeneous definitions of person-centred care in different concepts, cultures, care environments and contexts (De Silva, 2014). This heterogeneous definition is accompanied by vagueness in terms of the required quality indicators (Bru-Luna et al., 2021) and the finding that measurements assess different dimensions or components of person-centredness (Ekman et al., 2020; Elfstrand Corlin & Kazemi, 2024; van Dongen et al., 2023). Some measurements assess the same concepts but name them differently (Elfstrand Corlin & Kazemi, 2024). These findings imply how heterogeneous the concept is and how heterogeneously it is measured (De Silva, 2014), making a conceptual comparison of the assessments difficult (Edvardsson & Innes, 2010), especially when the conceptual underpinnings of measurements are not transparent, as reported in scientific publications (Ekman et al., 2020). The respective theoretical framework of an assessment influences and shapes how care is assumed and understood (Kazemi and Elfstrand Corlin, 2026). These challenges might explain why person-centred care implementations are less effective (Saragih et al., 2025).

The heterogeneous definitions and characteristics of person-centred care go along with challenges in the psychometric properties of measurements of person-centred care (van Dongen et al., 2023). In our literature search, various reviews reported different psychometric qualities of measurements assessing person-centred care. Fewer studies use validated person-centred care measurements to assess person-centred care outcomes (Pakkonen et al., 2021). Furthermore, the various measurements assess the perspectives of different stakeholders in the care of people living with dementia but often skip the perspective of the provider (Ahmed et al., 2019) and the person living with dementia (Edvardsson & Innes, 2010).

Another way to evaluate the implementation of person-centred care in nursing homes is to use person-centred care outcome measurements that are directly linked to the person-centred care concept implemented in nursing homes. We define outcomes on the conceptual level as outcomes such as autonomy and relationships. In the literature search, only a few included reviews addressed resident outcomes such as the quality of relationships with staff (McCabe et al., 2020). Outcomes such as relationships, autonomy, empowerment, respect, and dignity were not addressed in the included reviews.

Furthermore, the included reviews did not address intermediate outcomes, nor did they differ between intermediate outcomes and final endpoints. Intermediate outcomes can be defined as the mediation between the immediate outcome and the final endpoint and include aspects such as behaviours of the person living with dementia or quality of care (Hong & Oh, 2020). Intermediate outcomes could also be used as substitutes for final outcome measures or provide insights into the logic pathway (Jonas et al., 2018; Seuc et al., 2013; Velentgas et al., 2013) to impact person-centred dementia care-related resident-driven outcomes. However, intermediate outcomes can serve as implementation outcomes (Lengnick-Hall et al., 2022; Proctor et al., 2023) that are a relevant precondition for achieving the resident-reported outcome. Therefore, intermediate outcomes can also provide details about implementation feasibility, acceptance and/or facilitators/barriers since they are more influenced by care practices than are resident long-term endpoints (Damschroder et al., 2022).

### 5.2 Challenges in assessing person-centred care from an intervention perspective

When person-centredness is not assessed from a conceptual perspective, intervention outcomes can serve as proxy descriptors of person-centredness (Edvardsson & Innes, 2010). Therefore, the selection of outcomes and their measurements in intervention studies aims to detect change (Couch et al., 2020). However, several outcomes are influenced by the selected measurement approach (De Silva, 2014).

Nonetheless, the reviews included in our literature search present some challenges regarding outcomes and measurements. Our results illustrate that the effectiveness of person-centred care interventions is measured by heterogeneous outcomes that differ across studies. Furthermore, numerous outcomes are measured with diverse measurements in intervention studies, and most of those measurements are used only once. For example, for the outcome of cognitive impairment, we identified 63 different assessments that were used to measure this outcome. For agitation, we identified sixteen measurements to assess agitation, including the Cohen-Mansfield Agitation Inventory (CMAI) (Cohen-Mansfield, 1991), the Brief Agitation Rating Scale (BARS) (Finkel et al., 1993), the Pittsburgh Agitation Scale (PAS) (Rosen et al., 1994), and the Agitation Behaviour Mapping Instrument as cited by Kim and Park (2017). Couch et al. (2020) reported 40 different measurements to assess cognition as a widely used outcome for interventions. Measurements are also used in different studies for the measurement of different outcomes. For example, the Neuropsychiatric Inventory (NPI) (Cummings et al., 1994), which is criticized for its scoring, psychometric properties, and caregiver distress (Lai, 2014), is used to measure agitation, behaviour, well-being, and depression.

The heterogeneity of outcome measurements is characterized not only by standardized measurements but also by self-developed measurements (Zhao et al., 2021) and idiosyncratically adapted or inappropriate outcome measurements (Bird et al., 2016). Furthermore, heterogeneity is characterized by differences in the quality of the psychometric properties (Harper et al., 2022) or a lack of information about the psychometric properties of the measurements (Brownie & Nancarrow, 2013). The consequences of heterogeneous measurements of person-centred care outcomes are conceptual imprecision (Pakkonen et al., 2021) and challenges in drawing conclusions about the association between a respective domain and a specific outcome (Shier et al., 2014), especially in the context of confounders (Robinson et al., 2008).

Furthermore, studies that have not detected significant effects of person-centred care interventions might include outcome measures that are not adequate to detect changes associated with a specific intervention (Reilly & Harding, 2025). Several reviews report substantial heterogeneity in outcomes in the evaluation of several intervention types and studies (Couch et al., 2020; Hirt et al., 2021; Wong et al., 2024). This challenge is declared a limitation when researchers conduct a combination, comparison, or meta-analysis of the effects of several studies (Barbosa et al., 2017; Burgers et al., 2021; Wong et al., 2024).

A literature search revealed that heterogeneous individual resident measurements include outcomes designed mainly to assess behaviour, quality of life, cognitive function, and functional status. Our results also highlight significant heterogeneity in outcome measurement (Kelly et al., 2023). Key aspects of person-centredness according to Kitwood (1997), including psychological needs such as attachment, comfort, inclusion, identity, occupation, and love, are often not included in the description of the intervention and therefore not defined as relevant outcomes for people living with dementia. Reilly and Harding (2025) identified outcome items valued by people living with dementia, such as maintaining good relationships with relevant others or communicating with others, and highlighted that they do not agree with typically used outcomes. However, momentum towards more resident-centred, participatory evaluation approaches in the development and application of outcome measures has been growing over the past five years. Studies recommend the adoption of measurements that reflect the lived experiences and priorities of people living with dementia in nursing homes (Kelly et al., 2023; Reilly & Harding, 2025).

### 5.3 Challenges in assessing person-centred care from an organizational perspective

As described above, we addressed the relevance of the organizational perspective in the implementation of person-centred care. Implementation-related person-centred outcomes usually address outcomes from an organizational perspective that include the same challenges that we have mentioned above. However, unlike implementation-related outcomes, the organizational perspective focuses on established routines such as internal regulation. With respect to the assessment of person-centred care described above, we focused on the assessment of the policies and internal regulations of person-centred dementia care (Hoffmann et al., 2022). Nonetheless, this measurement has several challenges. The suitability of internal regulations is uncertain when person-centred dementia care is assessed. The literature (Hoffmann-Hoffrichter et al., 2025; Pot, 2022) discusses regulations of person-centred dementia care that differ from those types of regulations that are assessed in the Dementia Policy Questionnaire, for example. Furthermore, whether the existence of internal regulations on person-centred dementia care in a nursing home can be used to make a valid statement about the actual extent of implementation of this approach is unclear.

The evaluation of the Dementia Policy Questionnaire revealed that the measurement lacks adequate construct validity since it does not fully measure person-centred dementia care. Furthermore, the items of the questionnaire are too specific for the construct of person-centred dementia care. Additionally, the use of a dichotomous answer format is challenging since the person-centred care concept contains many nuances that cannot be answered solely with a dichotomous answer format.

## 6 Conclusions

**Fig. 1:**
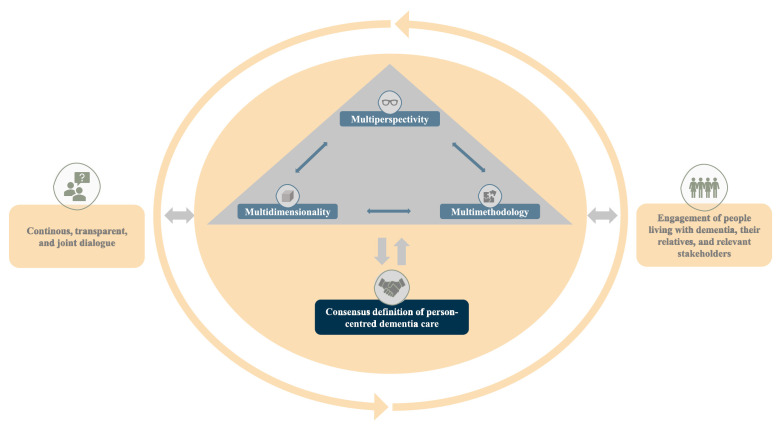
The process for a holistic measurement of person-centred dementia care.

In this discussion paper, we present details regarding the measurement of person-centred dementia care in nursing homes from conceptual, interventional, and organizational perspectives. We discussed the challenges associated with assessing person-centred care from each perspective. Based on our critical reflection, we frame our recommendations as conclusions where we address key aspects that are closely related to each other and that we deem necessary for suitable and holistic measurement of person-centred care in the future, including (1) a consensus on a definition of person-centred dementia care and (2) multidimensional, (3) multiperspective, and (4) multimethodological approaches that aim to capture the core of person-centred dementia care. All key aspects are consistent with and influenced by the engagement of people living with dementia, their relatives, and relevant stakeholders, as well as a continuous, transparent and joint dialogue for every key aspect. Our conclusions are shown in Fig. 1.

### 6.1 Consensus definition of person-centred dementia care

For a nursing home, the definition of person-centred care is based on the common understanding a nursing home has in relation to person-centredness. The common understanding of person-centred care within a nursing home is defined by reflexive processes that continue to evolve due to the carrying dynamics in a respective nursing home. Therefore, in both research and in nursing practice, the definition of person-centred dementia care can vary heterogeneously and, in particular, depend on the cultural context (Hoffmann-Hoffrichter et al., 2025). A definition of person-centred dementia care should furthermore adapt to societal changes in the health care system, such as multiculturalism and marginalized group needs, as well as requests from people living with dementia, their family caregivers and relevant stakeholders.

With respect to the measurement of person-centred care from conceptional, interventional, and organizational perspectives, the results of the literature search reveal that a succinct definition of person-centred care and its components as well as the provision and transparent reporting of an explicit conceptual framework is of utmost relevance.

### 6.2 Multidimensional measurements that assess person-centred dementia care

We assume that person-centred care is multidimensional and includes a dynamic interplay of various practices, such as moral, relational, communicative, and organizational practices, from several perspectives. Person-centred care contradicts the measurement of only one or a few aspects of this approach, e.g., the measurement of internal regulation or the physical environment, to make a valid statement about the implementation of person-centred care in a nursing home. Future research must focus on the development of person-centred care measurements that overcome the focus on single components of person-centred care. Elfstrand Corlin and Kazemi (2024) suggest reframing person-centred care measurements from an outcome to a continuous process. We agree with this suggestion and conclude the following: (1) the holistic measurement of person-centred care requires various measurements that provide insights into different aspects of person-centred care, and (2) person-centred care measures need to be constantly reflected on and feedback should be provided based on one’s own understanding of person-centred care to further develop person-centred care definitions and measurement. This approach lays the groundwork for obtaining a holistic picture of the implementation of person-centred care and for the further development of person-centred care definitions and measurement.

### 6.3 Multiperspective measurements that assess person-centred dementia care

We must consider various measurements that focus on different perspectives in general as well as the perspectives of different stakeholders to obtain a holistic picture of the implementation of person-centred care in a nursing home and to adequately assess the multidimensionality of person-centred care. We assume that people living with dementia can provide valid information about whether person-centred care is practised in direct interactions in a nursing home. Persons living with dementia, their relatives, associates and other relevant stakeholders must be included in research investigating the selection, development, and testing of measures through a participatory approach to identify and define appropriate person-centred measures in general, outcomes and outcome measures. This approach paves the way for the consistency of relevant and adequate outcomes and their measures in future intervention studies to overcome the challenge of detecting significant effects of person-centred care interventions. Given that the experience of person-centred care is understood as a subjective experience (Edvardsson & Innes, 2010), approaches are required to capture the preferences, beliefs, experiences and values of a person to approximate the core of person-centred care.

### 6.4 Multimethodological measurements that assess person-centred dementia care

We assume that the understanding and definition of person-centred care in a nursing home are specific to its context. Therefore, the multidimensionality, multiperspectivity, and heterogeneity of person-centred care contradict and contest standardized quantitative measurement of person-centred care. A single standardized quantitative measure cannot capture this respective understanding in a holistic manner. A combination of qualitative and psychometric valid and reliable quantitative methods is needed to obtain a complete picture of the implementation of a person-centred care approach and expand the reliability of person-centred care measures. Since no one measurement fits all when assessing person-centred care, the combination of measurements should include multiple standardized and narrative person-centred care measurements and outcome measures. Kazemi and Elfstrand Corlin (2026) propose a reflexive approach that includes the combination of patient-reported experience measures with reflective interviewing to explore how the meaning of patient experiences is co-constructed within contexts.

In summary, we suggest that person-centred care measurement must extend standardized quantification and account for individual understanding, the individual context in which person-centred care occurs, and the perspectives of people living with dementia, their relatives, and relevant stakeholders. These measurements are possible only through continuous, transparent and joint dialogue in which person-centred care is reflected upon individually, with the nursing home, and with the person living with dementia.

## Funding

The German Center of Neurodegenerative Diseases e.V. (DZNE) e.V. (grant number N/A) funded this study as well as manuscript draft. The funders were not involved in the research process or manuscript writing.

## Declaration of generative AI and AI-assisted technologies in the writing process

During the preparation of this work the authors used DeepL translator to improve the readability and language of the manuscript. After using this tool/service, the author(s) reviewed and edited the content as needed and take full responsibility for the content of the published article.

## CRediT authorship contribution statement

**Anna Louisa Hoffmann-Hoffrichter:** Writing – review & editing, Writing – original draft, Visualization, Validation, Supervision, Project administration, Methodology, Investigation, Formal analysis, Data curation, Conceptualization. **Rebecca Palm:** Writing – review & editing, Validation, Supervision. **Bernhard Holle:** Writing – review & editing, Validation. **Martina Roes:** Writing – review & editing, Validation, Supervision, Conceptualization.

## Declaration of competing interest

The authors declare that they have no known competing financial interests or personal relationships that could have appeared to influence the work reported in this paper.

## Data Availability

Access to the data is possible upon request to the German Center of Neurodegenerative Diseases e.V. (DZNE) e.V., site Witten, Germany. Please contact the data management of the German Center of Neurodegenerative Diseases e.V. (DZNE) e.V., site Witten (data-management-witten(at)dzne.de).
